# Functional Cognitive Disorder Presents High Frequency and Distinct Clinical Profile in Patients With Low Education

**DOI:** 10.3389/fnagi.2022.789190

**Published:** 2022-03-31

**Authors:** Wyllians Vendramini Borelli, Priscylla Nunes de Senna, Wagner Scheeren Brum, Artur Francisco Schumacher-Schuh, Eduardo R. Zimmer, Márcia Lorena Fagundes Chaves, Raphael Machado Castilhos

**Affiliations:** ^1^Cognitive and Behavioral Neurology Center, Serviço de Neurologia, Hospital de Clínicas de Porto Alegre, Porto Alegre, Brazil; ^2^Graduate Program in Biological Sciences: Pharmacology and Therapeutics, Universidade Federal do Rio Grande do Sul, Porto Alegre, Brazil; ^3^Graduate Program in Biological Sciences: Biochemistry, Universidade Federal do Rio Grande do Sul, Porto Alegre, Brazil; ^4^Departamento de Farmacologia, Universidade Federal do Rio Grande do Sul, Porto Alegre, Brazil

**Keywords:** cognitive complaint, subjective cognitive decline, dementia, Alzheimer’s disease, public health, subjective memory impairment, major depression

## Abstract

**Introduction:**

Functional Cognitive Disorder (FCD) is a non-degenerative, common cause of memory complaint in patients with high educational levels. FCD has been insufficiently described in individuals with low education. Here, we investigated the frequency of FCD among individuals with low education.

**Methods:**

We analyzed retrospectively all new referrals from primary care to a tertiary memory clinic from 2014 to 2021. Final diagnosis, diagnostic work-up, clinical and cognitive testing data were compared between FCD and other diagnoses, grouped as Neurodegenerative Disorders (NDD). A regression model was used to assess the effect of education on the diagnosis. Data is shown in Mean [SD].

**Results:**

A total of 516 individuals (70.76 [10.3] years) with low educational attainment (4.5 [3.94] years) were divided into FCD (146, 28.3%) and NDD. Compared with NDD, FCD patients showed lower age at presentation (66.2 [9.4] vs. 72.6 [10.2], *p* < 0.001), higher Mini-Mental State Examination (MMSE) scores (22.4 [6.2] vs. 14.7 [7.8], *p* < 0.001) and Geriatric Depression Scale (GDS) scores (7.4 [5.4] vs. 5.3 [3.7], *p* = 0.0001).

**Discussion:**

Surprisingly, FCD was the most frequent diagnosis in a low educational setting. However, education was not associated with FCD. Individuals presenting FCD showed a distinct clinical profile, including younger age and higher depressive scores. Strategies to identify FCD in primary care settings may benefit both patients and healthcare systems.

## Introduction

Functional cognitive disorder (FCD) is within the umbrella-term “functional neurological disorders.” It indicates the presence of a cognitive complaint not caused by a systemic or brain disease (Stone et al., 2015; McWhirter et al., 2020). FCD is a poor predictor of progressive cognitive impairment, and only a minority of individuals with FCD evolve to dementia (Jessen et al., 2020). However, this clinical entity is prevalent in memory clinics worldwide (Pennington et al., 2019), and it may exhibit different clinical characteristics.

A variety of neurological presentations have been described in patients with FCD. Cognitive complaints, especially memory, are commonly presented by patients with FCD, though not confirmed objectively. This inconsistency between the cognitive evaluation and the clinical interview is a strong indicator of a functional disorder (Ball et al., 2020). By definition, individuals with FCD exhibit cognitive complaints in the absence of detectable cognitive decline, while individuals with Mild Cognitive Impairment (MCI) phenotypically manifest objective impairment. Patients with FCD typically present to the consultation presenting with symptoms of depression or anxiety (Stone et al., 2015). Subjective memory complaints were also often described by these patients (Jessen et al., 2020), which is a prevalent clinical entity in memory clinics worldwide. Paradoxically, affective disorders associated with cognitive complaints may be both the etiology and a strong predictor of further cognitive decline (da Silva et al., 2013; Jessen et al., 2020). Finally, the complex relationship between psychological distress and neuropathological changes is somewhat controversial.

Studies of the epidemiology of FCD found substantially variable results. Overlapping definitions, heterogeneous diagnostic criteria, and diverse methodology to classify these individuals are sources of variability for measuring its prevalence (Ball et al., 2020). Previous studies suggest that FCD prevalence ranges from 10% to over 50% of diagnoses in different clinical settings (Pennington et al., 2015; Bharambe and Larner, 2018; Luck et al., 2018; Wakefield et al., 2018). Most of these studies were conducted in high-income countries, especially with higher levels of education, which does not correspond to the majority of patients living with dementia (Livingston et al., 2020). Educational attainment is an important proxy of cognitive reserve, and it plays a significant influence on the pathophysiology of cognitive decline (Stern, 2012). An individual’s level of education has also been described as a long-term protective factor for anxiety and depression (Bjelland et al., 2008; Dias et al., 2021). However, whether education and FCD are associated remains unclear. Herein, we aimed at identifying the frequency of FCD in a Brazilian tertiary memory clinic with a low education patient profile.

## Materials and Methods

### Study Design

A retrospective analysis was conducted with all new referrals to our tertiary memory clinic from January, 2014 to January, 2021. Individuals were referred by a general practitioner or family physician from the primary care setting of the Brazilian public health system (SUS, Sistema Único de Saúde). All patients that attended the memory clinic as a primary consultation were included in this study. This study was approved by the institution Ethics Committee under the IRB number 4.645.978.

### Data Collection

Patients underwent a routine evaluation comprising a semi-structured interview, cognitive screening evaluation, neurological examination, neuropsychological evaluation according to clinical indication, a neuroimaging exam (either a CT or MRI), and laboratory screening for potentially reversible causes of dementia (syphilis, HIV, B and C hepatitis serology, B12, folate, thyrotropin, creatinine, electrolytes levels, and total blood count). Then, they were classified into dementia syndromes according to the international diagnostic criteria as follows: MCI(Petersen et al., 1999), Alzheimer’s disease dementia (AD; McKhann et al., 2011), vascular dementia (VD; Sachdev et al., 2014), mixed dementia (AD and VD), and other less prevalent conditions [frontotemporal dementia (FTD; Rascovsky et al., 2011), Lewy bodies dementia, corticobasal syndrome, among others] (McKeith et al., 2017). Individuals with advanced stages of dementia, with indistinguishable clinical characteristics at disease onset, were classified as “Unspecified.” In order to distinguish and characterize FCD from other diagnoses, individuals with a diagnosis of any type of dementia were included in the Neurodegenerative Disorder group (NDD).

Functional Cognitive Disorder was defined according to previously published criteria (Ball et al., 2020), as follows: (1) one or more symptoms of cognitive impairment; (2) clinical evidence of internal inconsistency (discrepancy between a complaint and clinical judgment); (3) symptoms of impairment not explained by another medical condition; and (4) symptoms cause clinically substantial distress or impairment in social, occupational, or other important areas of function, or warrant medical evaluation. Individuals diagnosed with subjective cognitive decline were also included as FCD (Jessen et al., 2020). Importantly, FCD is a clinical entity that suggests a psychological/functional basis of cognitive complaint or even cognitive decline.

### Outcome Measures

Electronic records of all patients were evaluated for the following demographic and cognitive variables: date of appointment, age, sex, education, use of any substance (alcohol, tobacco, illicit drugs), previous and current pathologies, medication in use, the Mini-Mental State Examination (MMSE; Folstein et al., 1975), the Geriatric Depression Scale–15 item version (GDS; Yesavage et al., 1982), and the Functional Activities Questionnaire (FAQ; Pfeffer et al., 1982).

### Statistical Analysis

Frequencies and categorical variables were compared with Chi-squared tests. A logistic regression model was performed using age, education, and total MMSE scores as predictors of NDD. When appropriate, group comparisons between FCD, MCI, and NDD individuals were performed using analysis of variance with Tukey’s *post-hoc* test, and chi-square tests. We performed Bonferroni’s correction for multiple comparisons and *p*-values were considered significant at <0.05. Continuous variables were defined as mean ± standard deviation. Data analysis was performed using R 3.6.2 (R foundation for statistical computing, 2016), and variables with missing values above 9% were excluded. Comorbidities that were not described in the records were considered absent, or not diagnosed yet. Missing data are exhibited in the Supplementary Table 1.

## Results

Five hundred and sixteen (516) patients (mean age 70.76 ± 10.3 years, 61% females) were referred to the memory clinic from January 2014 to January 2021. The whole sample presented an average of 4.5 (± 3.94) years of education, including 71 (13.75%) illiterates, and mean MMSE score was 14.42 (± 8.17). Clinical comorbidities and use of substances are described in Table 1. Males presented increased alcohol use (Supplementary Table 3, *p* < 0.001).

**TABLE 1:** Demographic and clinical characteristics of patients that attended the memory clinic within the period of 2014–2021.

	Functional cognitive disorder (*n* = 146)	Mild cognitive impairment (*n* = 51)	Neurodegenerative disorders (*n* = 299)	Corrected *p*-value
Age, mean (SD)	66.2 (± 9.4)	73.7 (± 8.4)	72.4 (± 10.4)	**<0.0001**
Sex (F)	100 (68.5%)	29 (56.9%)	174 (58.2%)	0.87
Education, mean (SD)	5.6 (± 3.9)	5.3 (± 3.9)	4.9 (± 4.0)	0.17
Mini-mental state examination, mean (SD)	22.4 (± 6.2)	21.4 (± 4.4)	13.6 (± 7.6)	**<0.0001**
Geriatric depression scale–15 item, mean (SD)	7.4 (± 4.5)	3.5 (± 3.0)	5.7 (± 3.7)	**<0.0001**
Functional assessment questionnaire, mean (SD)	6.9 (± 7.6)	6.2 (± 5.7)	20.2 (± 8.4)	**<0.0001**
**Substance use, *n* (%)**				
Active smoking	13 (8.9%)	6 (11.8%)	22 (7.4%)	0.82
Active alcohol drinking	5 (3.4%)	3 (5.9%)	12 (4.0%)	0.55
**Comorbidities, *n* (%)**				
Hypertension	83 (56.8%)	34 (66.7%)	187 (62.5%)	0.37
Diabetes	33 (22.6%)	17 (33.3%)	83 (27.8%)	0.26
Dyslipidemia	31 (21.2%)	20 (39.2%)	75 (25.1%)	0.04
Malignancy	7 (4.8%)	3 (5.9%)	22 (7.4%)	0.65
Heart failure	5 (3.4%)	0	10 (2.9%)	0.57
Hypothyroidism	14 (9.6%)	5 (9.8%)	23 (7.7%)	0.69
Major depression	25 (17.1%)	3 (5.9%)	16 (5.4%)	**<0.0001**
**Medications, *n* (%)**				
Anti-hypertensive drug	79 (54.5%)	32 (62.7%)	170 (57.0%)	0.6
Antidepressant drug	69 (47.6%)	14 (27.5%)	106 (35.6%)	0.01
Antipsychotic drug	33 (22.8%)	3 (5.9%)	85 (28.5%)	0.0007
Acetylcholinesterase inhibitor	5 (3.4%)	4 (7.8%)	58 (39.7%)	**<0.001**
Benzodiazepines	20 (13.8%)	5 (9.8%)	34 (11.4%)	0.8

*FCD, functional cognitive disorder; MCI, mild cognitive impairment; NDD, neurodegenerative disorders. Bold represents statistically significant p-values.*

**FIGURE 1:**
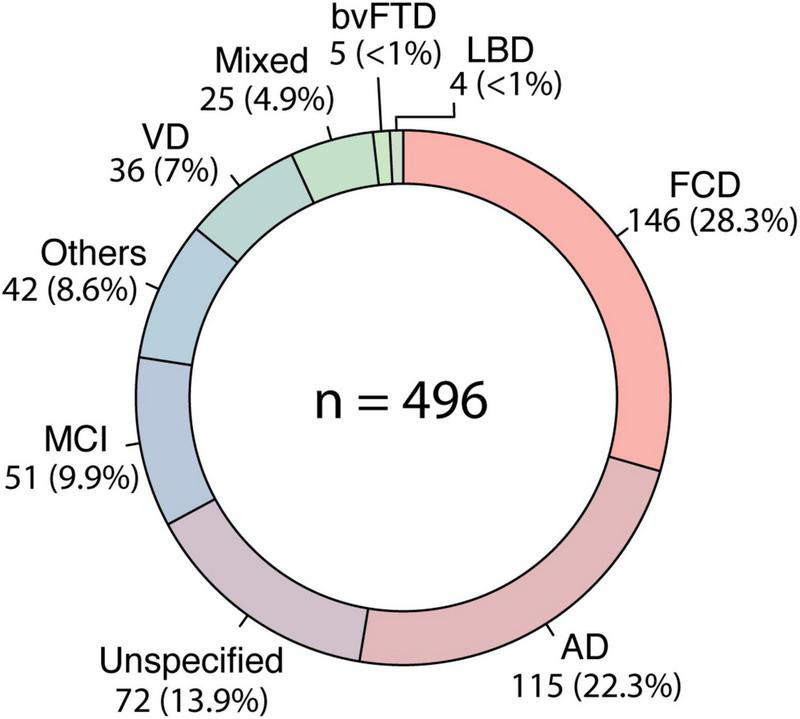
Frequency of diagnosis. The circle represents the frequency of diagnosis in our sample (*n* = 516), from 2014 to 2020. FCD, functional cognitive disorder; AD, Alzheimer’s disease; MCI, mild cognitive impairment; VD, vascular dementia; bvFTD, behavioral variant frontotemporal dementia; LBD, lewy bodies dementia.

Functional cognitive disorder (146, 28.3%) was the most common diagnosis, followed by AD dementia (115, 22.3%), MCI (51, 9.9%), VD (36, 7%) and mixed-type dementia (25, 4.9%) (Figure 1). Other less common diagnoses included behavioral variant FTD (5, 0.9%), Lewy bodies dementia (4, 0.7%), unspecified dementia (72, 13.9%) and others (42, 8.6%). Patients under investigation (19, 3.7%) were not included in this analysis.

**FIGURE 2:**
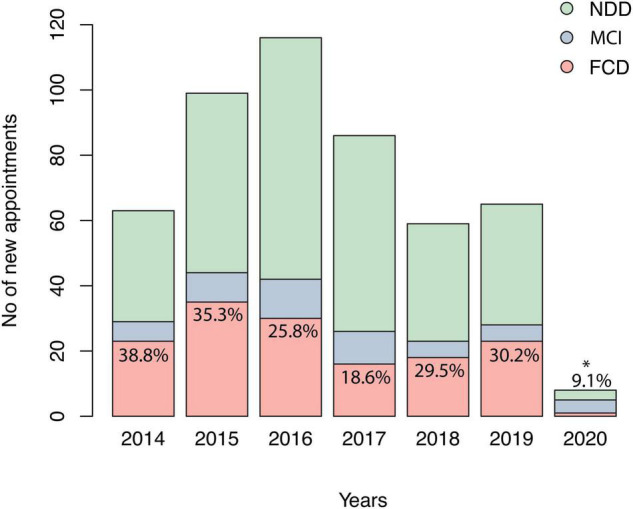
Number of new appointments in the memory clinic studied. Relative percentages represent the number of diagnoses of FCD divided by total new appointments of the corresponding year. FCD, functional cognitive disorder; MCI, mild cognitive impairment; NDD, neurodegenerative disorders. *Decrease in total number of appointments related to Coronavirus disease pandemic.

Ninety-three patients with FCD (63.7%) were diagnosed with a psychiatric disorder, namely major depressive disorder, anxiety or bipolar disorder, and 53 (36.3%) with Subjective Cognitive Decline. FCD accounted for an annual average of 16.79% of all referrals in this period, varying from 9.1% (2020, *n* = 1/11) to 38.84% (2014, *n* = 23/66) (Figure 2). Compared with NDD, patients with FCD presented higher MMSE and GDS and lower age and FAQ (Table 1). MCI also exhibited distinct characteristics when compared with FCD (Table 1). Though they presented similar MMSE scores, MCI was significantly older than FCD individuals, and had less depressive symptoms (*p* < 0.0001 for both).

Females presenting NDD were older and exhibited a higher frequency of major depression than males presenting NDD (Supplementary Table 3, *p* = 0.03 and *p* < 0.0001, respectively). Duration of symptoms, history of hypertension, diabetes, hypercholesterolemia and heart failure were similar between groups (*p* > 0.05), but the frequency of major depression was higher in FCD (*p* < 0.001). Cardiovascular and clinical comorbidities risk factors were similar between groups. It is also important to mention that five (5) FCD individuals were receiving an acetylcholinesterase inhibitor. In a logistic regression model, including age, education, and total MMSE, only age (*p* < 0.001) and total MMSE scores (*p* < 0.001) were predictors of FCD diagnosis (Table 2). The FAQ and GDS scores were not included in the regression analysis.

**TABLE 2:** Logistic regression model using diagnosis of neurodegenerative disorder as outcome.

Variable	Estimate	*z*-value	*p*-value	OR (95% CI)
Age	0.05	0.01	0.006	1.06 (1.03–1.08)
Education	0.09	0.03	<0.001	1.09 (1.02–1.17)
MMSE scores	−0.16	0.02	<0.001	0.84 (0.8–0.88)

## Discussion

Functional cognitive Disorder was the most frequent diagnosis among patients with low education, even more frequent than Alzheimer’s disease. Our sample consisted of individuals directly referred from primary care within the public health system, which represents the majority of individuals nationally. Although education was not a predictor of FCD, patients presenting FCD showed a distinct profile of clinical presentation.

Patients with low educational attainment are under-represented in studies of neurodegenerative diseases worldwide, even though most patients with dementia live in low and middle-income countries (Livingston et al., 2020). High level of education is a proxy of cognitive reserve, and it should be considered in studies of dementia and cognitive decline (Stern, 2012). Our findings indicate that FCD is also common in a low educational setting. The frequency we found was similar to observed in studies conducted in contexts with a higher level of education, which ranged from a quarter (Luck et al., 2018) to a half of patients consulting in a tertiary memory clinic (Bharambe and Larner, 2018). A complex range of reasons may be associated with this similarity. The contrast between cognitive evaluation and the clinical complaint is a hallmark of FCD, which is possibly not associated with brain areas connected to education or cognitive reserve (Ball et al., 2020). Moreover, primary care physicians from high and low educational settings may share similar doubts in diagnosing dementia. Besides, psychological distress is widely spread, but highly underdiagnosed in both settings (Dell’Osso et al., 2013; Lyu et al., 2017).

In fact, distinguishing FCD from cognitive symptoms of NDDs is challenging. Cell senescence has been increasingly associated with pathological brain aging, as its biological mechanisms may play a central role in elucidating the aging process and neuroinflammatory response (Chinta et al., 2015). Astrocytic and microglial senescence has been linked to age-associated inflammation and decreased neuroprotection (Gosselin and Rivest, 2018). Cognitive reserve, here proxied by education, has been described as a major neuroprotective factor during brain aging, but its underlying mechanisms are widely unclear. Low education may possibly reflect increased cell senescence process and early cognitive impairment due to many mechanisms (Verkhratsky et al., 2022). It is hypothesized that education may interact with glial cells that assume protective states in individuals with higher reserve. Besides, inflammaging is a well described phenomenon (Giunta et al., 2008) involving astrocyte senescence, microglial dystrophy (Shahidehpour et al., 2021) and ultimately neuronal integrity (Sikora et al., 2021). Plastic cell responses to brain pathology may involve dendritic pruning (Kirch and Gollo, 2021), but also astrocytes exhibit detrimental processes in synaptic transmission in cognitive aging (Sikora et al., 2021). Astrocytes may particularly contribute to neuroprotection, both stabilizing synapses and improving cell survival (Toricelli et al., 2021). Elucidating these mechanisms will be pivotal to identify biomarkers that distinguish functional from NDDs in subjective cognitive decline (McQuail et al., 2021). Further studies may focus on the neurobiological mechanisms of the interaction between low education and cell senescence phenotypically presenting as cognitive impairment.

Medical diagnosis of major depressive disorder was significantly higher in individuals with FCD when compared with other groups. It is well known that mood disorders are often associated with subjective cognitive complaints or objective cognitive decline (Perini et al., 2019). Besides, cognitive decline is described as a core symptom of depression, indicating that the impairment may also persist after the resolution of depressive symptoms and potentially worsen with repeated episodes (Rock et al., 2014; Semkovska et al., 2019). Furthermore, individuals with major depression share a similar pattern of brain atrophy than MCI (Zacková et al., 2021), possibly indicating an irreversibility of cognitive manifestations of depression. Referral of individuals with FCD to tertiary memory clinics may have several negative consequences, such as delaying of proper medical care and potential for worsening of symptoms, especially with regard to the risk of suicide, both impacting the public health system.

In a public health context, uncovering the mist around individuals with FCD is beneficial for both patients and healthcare systems. The correct identification of individuals with FCD diminishes the delay between diagnosis and treatment, which improves their quality of life and the clinical management of untreated mental disorders (Dell’Osso et al., 2013). This is especially important considering the referral process of public health systems, such as the Brazilian and the United Kingdom systems (Calil et al., 2020), in which the primary care physician is in charge of appointments with specialists. Besides, adequate referral to neurological tertiary care should include patients with a high probability of a NDD, mitigating the financial burden of unnecessary imaging and laboratory exams (Lyu et al., 2017). Potential factors underlying the unnecessary referral of FCD to a tertiary memory service include the overidentification of age-related cognitive decline, a lack of training in recognizing functional complaints by primary care professionals, and the stigma present in the elderly with memory complaints. In our study, individuals with FCD were predominantly middle-aged and presented high MMSE scores, which is in agreement with previous studies (Kroenke et al., 2003; Pennington et al., 2015; Bharambe and Larner, 2018; Wakefield et al., 2018). In addition, we did not identify differences between the groups regarding cerebrovascular risk factors. In Brazil, the frequency of those risk factors is frequent in middle-aged individuals, which may explain the similarity between groups (Dell’Osso et al., 2013). Additionally, a study found an association of poorer cardiovascular health and psychiatric disease (Lyu et al., 2017).

A few strategies may be implemented to optimize the identification and treatment of FCD in primary care and to decrease the number of referrals to a tertiary care level. Primarily, it is necessary to raise primary care physicians’ awareness that FCD is a common clinical entity with potential misdiagnosis with dementia (Ball et al., 2020). Besides, efficient, continuous training for primary care physicians may improve the identification of FCD. In addition, brief screening scales for mood disorders are available in many languages, and it may be useful for general practitioners, such as 2-question screening scales for symptoms of depression (Kroenke et al., 2003) and anxiety (Kroenke et al., 2007). Lastly, implementing and disclosing the usage of “dementia hotlines” may help identify FCD by general practitioners. Telehealth consultations have been described as critical in improving healthcare in small cities and avoiding unnecessary referrals (Harzheim et al., 2016; Marcolino et al., 2016). Also, recent advances in the development of blood biomarkers promise to optimize referral processes in public health systems, owing mainly to their differential diagnosis capabilities (Karikari et al., 2020; Palmqvist et al., 2020). Further studies may provide evidence in increasing the early identification of FCD in primary care settings and its impact in public health measures.

There are some limitations in this study. First, its retrospective design contributed to the missing data for many variables, such as education, MMSE and GDS, which may reduce the internal validity of the results. Second, as a tertiary service belonging to a public health system lacking specialists, patients with advanced stages of disease may have been referred preferentially, which may hamper the assessment of differences in cognitive tests between FCD and NDD groups. Missing values may also introduce bias, as they were not computed in the analysis. Lastly, it is important to mention that 2020 was atypical because of the COVID-19 pandemic and lockdown measures, which reduced the referral to all specialties, including neurology.

In conclusion, FCD is a common clinical entity among individuals with low educational setting, totaling around one-third of tertiary care referrals. Patients that presented with FCD were younger, showed higher MMSE and higher depressive scores than NDD individuals. Strategies to identify FCD in primary care settings may benefit both patients and healthcare systems, including long-term training for physicians and implementing dementia hotlines.

## Data Availability Statement

The raw data supporting the conclusions of this article will be made available by the authors, without undue reservation.

## Ethics Statement

The studies involving human participants were reviewed and approved by the Comitê de Ética em Pesquisa do Hospital de Clinicas de Porto Alegre. Written informed consent for participation was not required for this study in accordance with the national legislation and the institutional requirements.

## Author Contributions

All authors listed have made a substantial, direct, and intellectual contribution to the work, and approved it for publication.

## Conflict of Interest

The authors declare that the research was conducted in the absence of any commercial or financial relationships that could be construed as a potential conflict of interest.

## Publisher’s Note

All claims expressed in this article are solely those of the authors and do not necessarily represent those of their affiliated organizations, or those of the publisher, the editors and the reviewers. Any product that may be evaluated in this article, or claim that may be made by its manufacturer, is not guaranteed or endorsed by the publisher.
