# Supplementary material for: Functional Cognitive Disorder Presents High Frequency and Distinct Clinical Profile in Patients With Low Education
Source: Front Aging Neurosci. 2022 Mar 31;14:789190. doi: 10.3389/fnagi.2022.789190 (PMC9011344; doi:10.3389/fnagi.2022.789190)
Supplement: Supplementary file 1 [file Data_Sheet_1.docx]

**Supplementary material**

Supplementary table 1. Missing values for each variable included in the study.

|  | **FCD**  **(n = 146)** | **NDD**  **(n = 350)** |
| --- | --- | --- |
| Age, mean (SD) | 0 | 0 |
| Sex (F) | 0 | 0 |
| Education, mean (SD) | 0 | 0 |
| Mini-mental State Examination, mean (SD) | 3 (2.1%) | 7 (2.0%) |
| Geriatric Depression Scale - 15 item, mean (SD) | 12 (8.2%) | 30 (8.5%) |
| Functional Assessment Questionnaire, mean (SD) | 14 (9.6%) | 30 (9.7%) |

FCD: Functional Cognitive Disorder; NDD: Neurodegenerative Disorder

Supplementary table 2. Descriptive analysis of every subgroup of dementia syndrome.

|  | **Functional Cognitive Disorder**  **(n = 146)** | **Alzheimer's disease**  **(n = 115)** | **Mixed type**  **(n = 25)** | **Vascular dementia**  **(n = 36)** | **Other types of dementia**  **(n = 174)** | **Corrected p-values** |
| --- | --- | --- | --- | --- | --- | --- |
| Age, Mean (SD) | 66.2 (±9.4) | 75.6 (±8.9) | 78.9 (±6.0) | 72.3 (±9.5) | 69.7 (±10.6) | **< 0.0001** |
| Sex (F) | 100 (68.5%) | 74 (64.3%) | 17 (68.0%) | 16 (44.4%) | 96 (55.2%) | **0.026** |
| Education, mean (SD) | 5.6 (±3.9) | 5.0 (±4.0) | 4.6 (±3.6) | 4.5 (±2.9) | 5.0 (±4.2) | 0.44 |
| Time of symptom onset, mean (SD) | 36.1 (±42.9) | 44.6 (±36.0) | 44.9 (±39.3) | 29.9 (±31.6) | 43.5 (±54.1) | 0.32 |
| Mini-mental state examination mean, (SD) | 22.4 (±6.2) | 12.4 (±7.1) | 10.8 (±7.1) | 15.4 (±4.9) | 16.7 (±8.2) | **< 0.0001** |
| Geriatric depression scale, mean (SD) | 7.4 (±4.5) | 4.6 (±3.4) | 6.9 (±3.5) | 7.7 (±4.3) | 5.0 (±3.5) | **< 0.0001** |
| Functional activities questionnaire, mean (SD) | 6.9 (±7.6) | 21.4 (±7.8) | 21.6 (±6.5) | 20.3 (±8.8) | 13.2 (±9.8) | **< 0.0001** |
| **Substance use, n (%)** | | | | |  |  |
| Active Smoking | 13 (8.9%) | 9 (7.8%) | 0 (0.0%) | 2 (5.6%) | 17 (9.8%) | 0.23 |
| Active alcohol drinking | 5 (3.4%) | 4 (3.5%) | 1 (4.0%) | 1 (2.8%) | 9 (5.2%) | 0.48 |
| **Comorbidities, n (%)** | |  |  |  |  |  |
| Hypertension | 83 (56.8%) | 65 (56.5%) | 20 (80.0%) | 29 (80.6%) | 107 (61.5%) | 0.018 |
| Diabetes | 33 (22.6%) | 21 (18.3%) | 11 (44.0%) | 18 (50.0%) | 50 (28.7%) | **0.0008** |
| Dyslipidemia | 31 (21.2%) | 26 (22.6%) | 9 (36.0%) | 8 (22.2%) | 52 (29.9%) | 0.26 |
| Malignancy | 7 (4.8%) | 9 (7.8%) | 2 (8.0%) | 2 (5.6%) | 12 (6.9%) | 0.84 |
| Heart failure | 5 (3.4%) | 1 (0.9%) | 1 (4.0%) | 3 (8.3%) | 5 (2.9%) | 0.18 |
| Hypothyroidism | 14 (9.6%) | 12 (10.4%) | 0 (0.0%) | 3 (8.3%) | 13 (7.5%) | 0.52 |
| Major depression | 25 (17.1%) | 4 (3.5%) | 3 (12.0%) | 1 (2.8%) | 11 (6.3%) | **0.0008** |
| Anti-hypertensive drug | 79 (54.5%) | 51 (44.3%) | 21 (84.0%) | 29 (80.6%) | 101 (58.4%) | **< 0.0001** |
| Antidepressant drug | 69 (47.6%) | 38 (33.0%) | 13 (52.0%) | 17 (47.2%) | 52 (30.1%) | 0.005 |
| Antipsychotic drug | 33 (22.8%) | 30 (26.1%) | 6 (24.0%) | 15 (41.7%) | 37 (21.4%) | 0.15 |
| Anticholinesterase inhibitor | 5 (3.4%) | 34 (29.5%) | 4 (16%) | 2 (5.5%) | 18 (10.3%) | **< 0.0001** |
| Benzodiazepines | 20 (13.8%) | 8 (7.0%) | 4 (16.0%) | 4 (11.1%) | 23 (13.3%) | 0.36 |

Supplementary table 3. Group differences according to different genders

|  | **FCD**  **(n = 146)** | | | **Neurodegenerative disorders**  **(n = 350)** | | |
| --- | --- | --- | --- | --- | --- | --- |
|  | **Females**  **(n = 100)** | **Males**  **(n = 46)** | sig. | **Females**  **(n = 203)** | **Males**  **(n = 147)** | sig. |
| Age, mean (SD) | 66.3 (±9.5) | 66.0 (±9.3) | 0.83 | 74.1 (±9.5) | 70.6 (±10.7) | **0.03** |
| Education, mean years (SD) | 5.4 (±4.0) | 6.1 (±3.6) | 0.29 | 4.7 (±3.8) | 5.3 (±4.2) | 0.25 |
| Time of symptom onset, mean (SD) | 34.1 (±43.5) | 40.5 (±41.9) | 0.052 | 41.8 (±35.2) | 43.6 (±56.6) | 0.26 |
| Mini-mental state examination mean, (SD) | 22.3 (±6.0) | 22.5 (±6.8) | 0.62 | 14.3 (±7.2) | 15.3 (±8.4) | 0.13 |
| Geriatric depression scale, mean (SD) | 7.3 (±4.4) | 7.4 (±4.8) | 0.98 | 5.2 (±3.8) | 5.4 (±3.5) | 0.41 |
| Functional activities questionnaire, mean (SD) | 6.0 (±7.8) | 8.7 (±7.0) | 0.39 | 18.0 (±9.4) | 17.7 (±9.7) | 0.83 |
| **Substance use, n (%)** | | |  |  | |  |
| Active Smoking | 8 (8.0%) | 5 (10.9%) | 0.135 | 17 (8.4%) | 11 (7.5%) | 0.15 |
| Active alcohol drinking | 3 (3.0%) | 2 (4.3%) | **< 0.0001** | 6 (3.0%) | 9 (6.1%) | **< 0.0001** |
| Hypertension | 56 (56.0%) | 27 (58.7%) | 0.86 | 130 (64.0%) | 91 (61.9%) | 0.74 |
| Diabetes | 22 (22.0%) | 11 (23.9%) | 0.83 | 51 (25.1%) | 49 (33.3%) | 0.095 |
| Dyslipidemia | 22 (22.0%) | 9 (19.6%) | 0.83 | 58 (28.6%) | 37 (25.2%) | 0.54 |
| Malignancy | 6 (6.0%) | 1 (2.2%) | 0.43 | 8 (3.9%) | 17 (11.6%) | 0.15 |
| Heart failure | 3 (3.0%) | 2 (4.3%) | 0.65 | 6 (3.0%) | 4 (2.7%) | 1.0 |
| Hypothyroidism | 10 (10.0%) | 4 (8.7%) | 1.0 | 21 (10.3%) | 7 (4.8%) | 0.072 |
| Major depression | 18 (18.0%) | 7 (15.2%) | 0.81 | 19 (9.4%) | 0 (0.0%) | **< 0.0001** |
| Anti-hypertensive drug | 54 (54.0%) | 25 (55.6%) | 1.0 | 117 (57.6%) | 85 (58.2%) | 1.0 |
| Antidepressant drug | 48 (48.0%) | 21 (46.7%) | 1.0 | 79 (38.9%) | 41 (28.1%) | 0.040 |
| Antipsychotic drug | 21 (21.0%) | 12 (26.7%) | 0.52 | 44 (21.7%) | 44 (30.1%) | 0.081 |
| Benzodiazepines | 14 (14.0%) | 6 (13.3%) | 1.0 | 24 (11.8%) | 15 (10.3%) | 0.73 |
